# On-site or off-site treatment of medical waste: a challenge

**DOI:** 10.1186/2052-336X-12-68

**Published:** 2014-04-16

**Authors:** Hassan Taghipour, Taher Mohammadyarei, Mohamad Asghari Jafarabadi, Ahmad Asl Hashemi

**Affiliations:** 1 Department of Environmental Health Engineering, Tabriz University of Medical Sciences, Tabriz, Iran; 2 Student Research Committee, Department of Environmental Health Engineering, Tabriz University of Medical Sciences, Tabriz, Iran; 3 Medical Education Research Center, Department of Statistics and Epidemiology, Tabriz University of Medical Sciences, Tabriz, Iran

**Keywords:** Medical waste, Treatment, AHP, On-site, Off-site, A challenge

## Abstract

Treating hazardous-infectious medical waste can be carried out on-site or off-site of health-care establishments. Nevertheless, the selection between on-site and off-site locations for treating medical waste sometimes is a controversial subject. Currently in Iran, due to policies of Health Ministry, the hospitals have selected on-site-treating method as the preferred treatment. The objectives of this study were to assess the current condition of on-site medical waste treatment facilities, compare on-site medical waste treatment facilities with off-site systems and find the best location of medical waste treatment. To assess the current on-site facilities, four provinces (and 40 active hospitals) were selected to participate in the survey. For comparison of on-site and off-site facilities (due to non availability of an installed off-site facility) Analytical Hierarchy Process (AHP) was employed. The result indicated that most on-site medical waste treating systems have problems in financing, planning, determining capacity of installations, operation and maintenance. AHP synthesis (with inconsistency ratio of 0.01 < 0.1) revealed that, in total, the off-site treatment of medical waste was in much higher priority than the on-site treatment (64.1% versus 35.9%). According to the results of study it was concluded that the off-site central treatment can be considered as an alternative. An amendment could be made to Iran’s current medical waste regulations to have infectious-hazardous waste sent to a central off-site installation for treatment. To begin and test this plan and also receive the official approval, a central off-site can be put into practice, at least as a pilot in one province. Next, if it was practically successful, it could be expanded to other provinces and cities.

## Introduction

Medical waste includes all of waste that is produced in the course of health protection, immunization, diagnosis, medical treatment of human beings or animals, scientific research and related laboratories [1-6]. Between 75% and 90% of the waste produced by health-care providers is non-risk or general health-care waste, the remaining 10-25% of health-care waste is regarded as hazardous-infectious and may create a variety of health risks [2,7]. In the USA, about 15% of total hospital waste is considered as infectious waste. But in India this could range from 15% to 35% and in Iran about 29.89% depending on the total amount of waste produced [8,9]. The rate of generation of waste in Iran was reported 2.71-4.45 Kg/bed-day but in other countries that varies from 0.84 -7 Kg/bed-day [2,8,10-15].

Basic principles of medical waste management include preventing and/or minimizing waste production, appropriately segregating general medical waste from hazardous-infectious medical waste, sending general medical waste to the municipal waste stream for final disposal and treating hazardous-infectious medical waste carefully using special methods [2,8,16-18]. For preventing and/or minimizing medical waste, implementation of certain policies and practices including the following ones can significantly help: choosing supplies and goods that are less wasteful or less dangerous, using physical rather than chemical cleaning methods, centralized purchasing of hazardous chemicals, monitoring chemical flows, ordering relatively small quantities rather than large amounts at one time, using the oldest batch of a product first, checking the expiry date of all products at the time of delivery, properly segregating general waste and hazardous-infectious waste [2,8]. But, the most important step in waste minimization of medical waste is appropriate segregation of hazardous-infectious waste from general waste. The segregation process should be carried out by waste producers. Segregating and sending the general medical waste to municipal waste disposal system can reduce at least about 70% of quantity of total generated waste and, as a consequence, its related difficulties and problems. One of the practical ways for segregation is colour-coded method. However, the hazardous-infectious medical waste needs special attention for treatment and final disposal [2-4,9,19,20]. Treating the hazardous-infectious medical waste can be carried out on-site or off-site of health-care facilities [2,8]. A lot of studies have been carried out in different countries and also Iran about characterization, regulation, management and treatment of medical waste [4,5,7,8,11,14,15,21-25]. Also some of studies have been carried out in comparison of on-site and offsite (central) facilities and determining their advantages and disadvantages for medical waste treatment [17,26-28]. Nevertheless, the selection between on-site and off-site methods for treatment location of medical waste is a controversial subject especially in Iran.

Currently in Iran, due to policies of Health Ministry, the hospitals (and other major producers of medical waste) have selected on-site treating method as the preferred treatment. Because, according to Act 64 in Iran’s Medical Waste Management Regulations, all waste producers in middle-sized and large cites are responsible for treating hazardous-infectious waste and converting it into general waste in on-site facilities. Only after on-site pre-treatment of medical waste will the municipality takes the responsibility for off-site transport of waste to the final disposal site. Nevertheless, according to the same Act of the regulation, small cities and villages are allowed to use off-site (central) facilities for treating their hazardous-infectious waste [29]. Based on Act 65 of Medical Waste Management Regulations in Iran, minor medical waste producers (physician's office, dental clinics, acupuncturists, chiropractors, small clinics, diagnostic laboratories) can use off-site (central or regional) facilities for treating their hazardous-infectious medical waste [29]. Another option for the minor medical waste producers is to send their waste to on-site establishment of neighboring hospitals [29].

In the meantime, there are great concerns about operation and maintenance conditions of on-site medical waste treatment facilities in hospitals. Therefore, the primary objective of this study was to assess the current condition of on-site medical waste treatment facilities in the country, compare on-site medical waste treatment facilities with off-site systems and find the best location by employing Analytical Hierarchy Process (AHP). In addition, this study aimed to make some practical recommendations on medical waste treatment for improving the current situation.

## Methodology

In the beginning of the study for assessing current on-site medical waste treatment facilities, 4 out of 31 provinces in Iran including East Azerbaijan, Tehran, Isfahan and Gilan were selected to participate in the survey. The selection of provinces was done in such a way to cover virtually different geographies, climates, economies, cultures. Then, 10 active hospitals with on-site medical waste treatment facilities were selected in each province (totally 40 hospitals) for assessing their current conditions. The selection of hospitals was carried out in such a way to cover various categories of hospitals (i.e., governmental, educational, university, private, NGO and military) and sizes. Site visits (observational method) with completing checklist were conducted in all selected hospitals to gather the basic information and assess current working conditions of on-site medical waste treatment facilities. Then, to compare on-site and off-site medical waste treatment facilities, based on the experts’ perspective Analytical Hierarchy Process (AHP) was employed. Different decision-making tools have been developed for application in the environmental field such as the Matrix Method, the AHP and the Electre III method. AHP is one of the most practical multiple choice decision making techniques among the available methods widely used [24,28]. It should be explained, currently there is not any installed off-site medical waste treatment facilities in country. So the direct comparison of two systems was not possible during this study.

### Criterion variables

Twenty two criterion variables for on-site treatment and off-site treatment were chosen for the Analytical Hierarchy Process. Then, by brainstorming high-level experienced experts (and also considering previous experiences, literature review) thirteen final criterion variables (among twenty two primary criterion) for both on-site and off-site treatment was selected for calculation of treatment location (as listed in Table 1). The high-level experienced experts include academic staffs of environmental health engineering departments who have experience and research in medical waste and also the managers, technical staffs and experts of hospitals that have duty in medical waste treatment and disposal. A survey was carried out to validate the selection criteria. The survey results were incorporated in finalization of the list of criterion variables [17].

**Table 1:** List of final identified criterion variables for on-site and off-site treatment options for Analytical Hierarchy Process

**No**	**Criterion variables**	**Abbreviation**
1	Capital, maintenance and operation costs	(CMOC)
2	Transportation cost of waste	(TCW)
3	Costs and problems of air and wastewater treatment	(CPAW)
4	Energy requirements	(ER)
5	Reliability and ease of operation	(REO)
6	Feasibility of treating medical waste of minor medical producers	(FTMMP)
7	Feasibility of treating medical waste of the surrounding area (cities and villages)	(FTMSA)
8	Need for skilled operators	(NSO)
9	Required space	(RS)
10	Continuous performance and monitoring the system	(CPMS)
11	Occupational risks in treatment site	(ORTS)
12	Environmental and health risks posed by transportation	(EHRT)
13	Compliance with laws and regulations	(CLR)

### Analytical Hierarchy Process

All the analysis was performed using Expert Choice 11 software (Expert Choice Inc., Arlington, Virginia, USA). The input data for the analysis was the weighted means resulted from the primary evaluation of the panel experts, which was calculated in Microsoft Excel 2007 software. Analytical Hierarchy Process (AHP) technique was used in two steps to prioritize the objects; it prioritized the criteria in the first step and alternative locations in the second step. In the AHP procedure, as one of multiple decision-making techniques, Eigen values and eigenvectors were computed based on the input data matrix; afterwards, the priority weights were computed to rank the criteria and alternatives. In this procedure, the inconsistency ratio was calculated to investigate consistency of ranking made by the experts. This index ranged between zero (complete consistency) and one (complete inconsistency) and the values lower than 0.1 indicated a reasonable level of ranking consistency and hence confirmed the results of prioritizing the objects. The results of the analysis were presented based on absolute and normalized weights; the absolute weights ranging between 0 and 1 with higher weights showed the higher rank and higher priority of the objects. To normalize the weights, the maximum weight would be transferred to one and other weights would change correspondingly.

## Results and discussion

### Current condition of medical waste treatment in on-site installations

One of the first and most important steps in making an effective decision for selection of location treatment site is to assess current experiences. Due to not using off-site medical waste treatment system in Iran, only on-site system was assessed for determining the current condition.

The survey on the kind of treatment technologies which have been employed in the on-site installations of the studied hospitals indicated that most of used methods included autoclave, hydroclave, chemical treatment and incinerator successively. The result of site visiting, completing checklist and gathering the related data for operating and maintaining on-site medical waste treatment systems are presented in Table 2. As indicated that table, capital cost of the used land and treating equipment was considerable in each hospital (about 85500 $). By applying the operation and maintenance cost in a long term, that will be even more noticeable. Selecting and ordering treating equipment is generally carried out without primary study of the amount of generated medical waste in each hospital and also considering the capacity of chosen devices. Most of the hospitals (82.5%) were equipped only with one treatment equipment, if the system found any technical problem, all the hazardous-infectious would be sent out without any treatment. In other 17.5% of hospitals which had parallel treating devices, the extra treating capacity was almost zero. Each on-site treatment facility needed at least two fulltime highly skilled technicians only for operating treating equipment. Nevertheless, allocation of two highly skilled technicians with current financial problems of hospitals almost was not practical. On the other hand, not allocation of proficient operators itself could cause the increase of operation and maintenance costs in the long run. According to the result of this study in 40 on-site medical waste treatment facilities in 4 provinces of Iran, about 32.5% of hospitals had difficulties in preparing spare parts of systems. It should be explained that most of on-site medical waste treatment facilities were recently installed. Probably, over time and by aging of facilities and treating systems, those operating and maintaining problems would be more noticeable in future. Almost none of 40 studied hospitals (with on-site treatment facilities) accepted and treated hazardous-infectious waste of minor medical waste producers of the city or the medical waste generated in the surrounding small cities and villages. Therefore, in the current condition, almost all minor medical waste producers sent their hazardous-infectious waste along with municipal waste stream without any treatment for final disposal. Because as indicated in Table 2, the major medical waste generators themselves had many problems with their on-site medical waste treatment systems due to high capital cost and also operation and maintenance problems and costs.

**Table 2:** Summary of assessing current on-site medical waste treatment systems in the studied area (40 hospitals)

**No**	**Surveyed subject**	**Result in the studied hospitals**	
1	Average used land (m^2^) in each hospital	About 86.25 m^2^	
2	Average cost per square meter (US $)	About 585 $	
3	Average capital cost of land per on-site facilities (US $)	About 50500 $	
4	Average capital cost per treating equipment (US $)	About 35000 $	
5	Using special foundation for installing treating equipment	Yes (55%)	No (45%)
6	Selected treating equipment from internal producers or from abroad	Internal (67.5%)	Abroad (33.5%)
7	Reporting any problem regarding availability of spare parts and maintenance of system	Yes (32.5%)	No (67.5%)
8	Average working hours per day	About 6 h	
9	Required skilled operators	At least 2	
10	Allocation of highly skilled operators for treating equipment	Yes (0%)	No (100%)
11	Selecting treating equipment capacity according to previous determination of the amount of medical waste	Yes (0%)	No (100%)
12	Having parallel treating equipment for emergency conditions (phasing out of the system)	Yes (17.5%)	No (82.5%)
13	Reliability of treating equipment according to self statement of operators	Yes (72.5%)	No (27.5%)
14	Using air pollution control system for treating equipment (incinerators)	No (100%)	
15	Accepting and treating medical waste of minor medical producers in the city	Yes (0%)	No (100%)
16	Accepting and treating medical waste of the surrounding cities and villages	Yes (0%)	No (100%)
17	Management quality of on-site facilities space from health viewpoint	Good (62.5%)	Bad (37.5%)

### Comparing on-site and off-site treatment of medical waste by Analytical Hierarchy Process (AHP)

**Figure 1:**
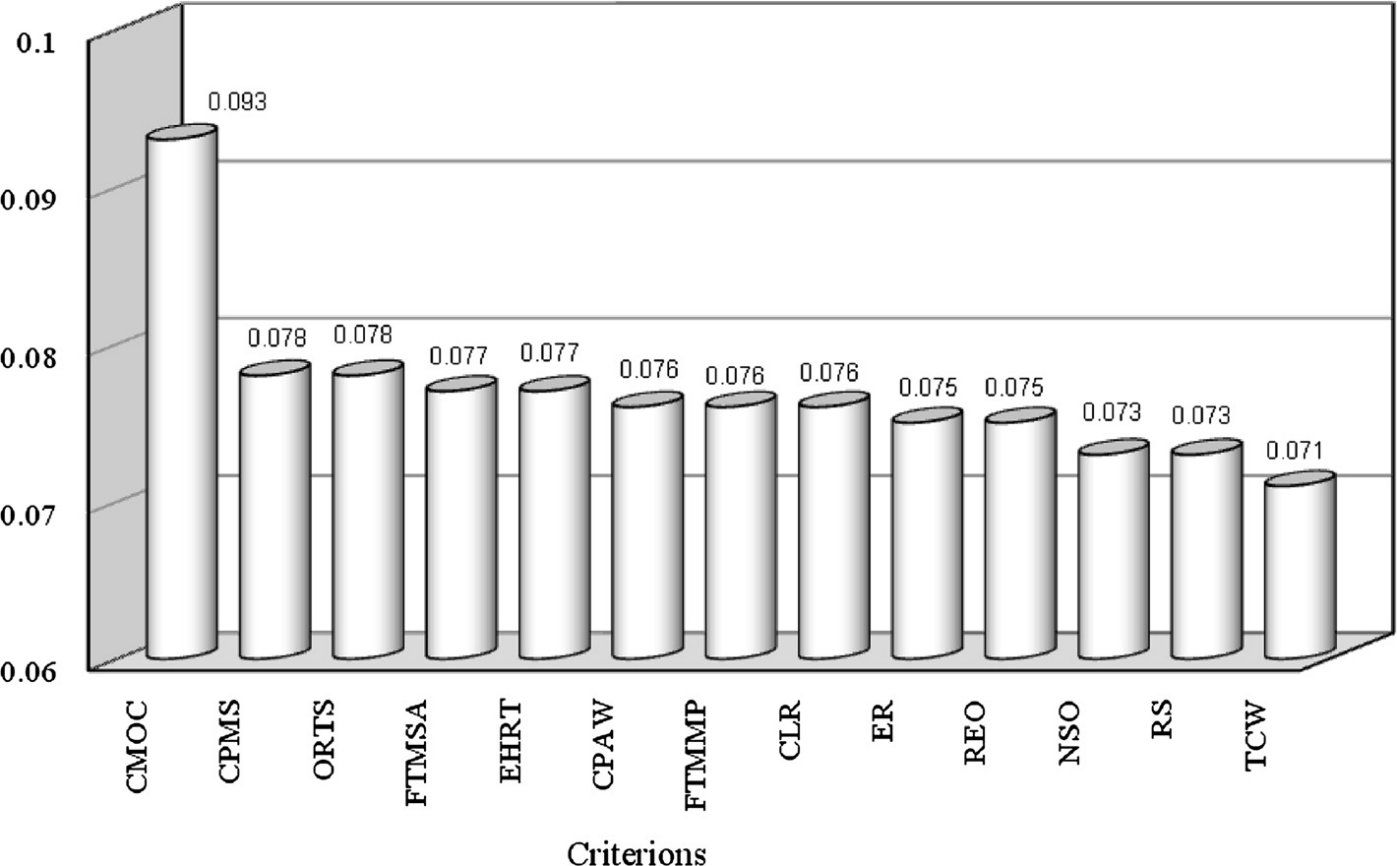
Priorities of creation with respect to goal: on-site or off-site treatment (inconsistency = 0.00612 with 0 missing judgments).

**Figure 2:**
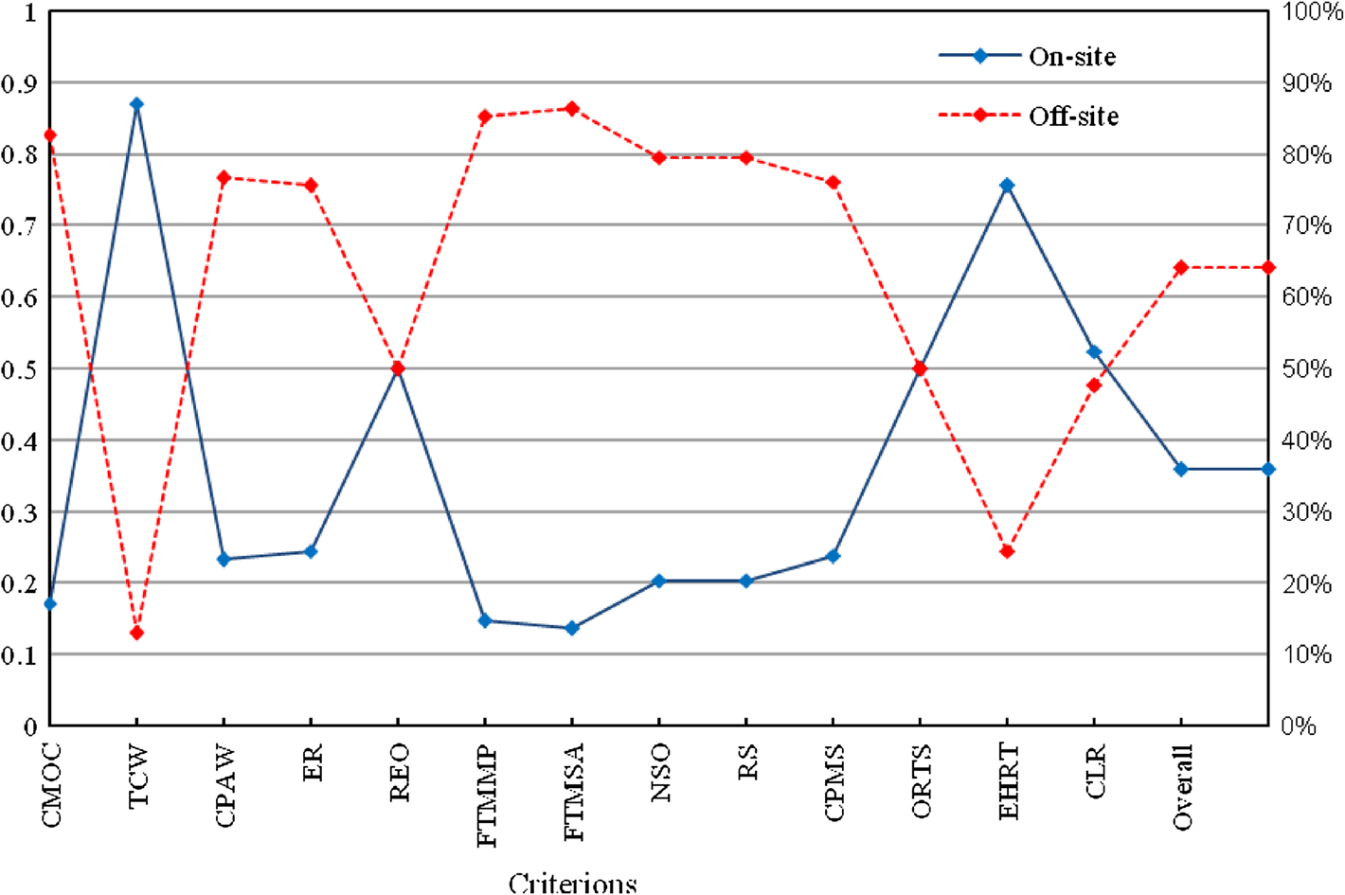
Comparing on-site with off-site treatment of medical waste in selected criteria and overall by Analytical Hierarchy Process (with inconsistency ratio of 0.01 < 0.1).

AHP presented results in several steps. First, prioritizing the criterion variables in which capital, maintenance and operation costs (CMOC = 0.093), continuous performance and monitoring the system (CPMS = 0.078) and occupational risks in treatment site (ORTS = 0.078) were prioritized as three first variables with the highest level of importance and need for skilled operators (NSO = 0.073) required space (RS = 0.073) and transportation cost of waste (TCW = 0.071) as three variables with lower level of importance (Figure 1). In this step, the inconsistency ratio (ICR) was 0.00612 with zero missing judgment, which confirmed the consistency of ranks suggested by experts. In the next step, for each criterion, on-site versus off-site treatment of medical waste was compared and prioritized. In each case, the ICR < 0.1 showed acceptable level of ranking consistency. Also in this step, the results showed that, for CMOC, CPAW, ER, FTMMP, FTMSA, NSO, RS and CPMS criterion variables, off-site treatment was at higher level of priority (Figure 2). But, for transportation cost of waste (TCW) and environmental and health risks posed by the transportation (EHRT) criteria, on-site treatment had higher importance. Also for CLR criterion, although a higher weight was observed in the side of on-site treatment of medical waste, but the difference of weight between on-site and off-site was not considerable. In addition, there were similar weights for both on-site and off-site treatment in REO and ORTS criterion variables. Finally, as presented in Figure 2, AHP synthesized the above mentioned results (with ICR = 0.01 < 0.1) and revealed that, in total, the off-site treatment of medical waste was in much higher priority than the on-site treatment (64.1% vs. 35.9%). Therefore, in most of the criterion variables and in total, the results confirmed the off-site treatment of medical waste as a better option. About treatment technology options it should be explained, by considering the previous unsuccessful experience of on-site incineration in Iran, current medical waste management regulations in the country [8,29] and the results of Analytical Hierarchy Process in this study (which was not presented in detail in this manuscript), the preferred treatment option was autoclave or hydroclave. In comparison with other researches, the results of this study was in agreement with finding of Karagiannidis et al. 2010 in Greece and also Oakan 2013 in Turkey which concluded respectively, a centralized (off-site) autoclave or hydroclave and the off-site sterilization technique as best option for medical waste treatment [27,28]. In the meanwhile Yerabandi et al. 1998 in Canada according to the Criterion Function approach and Analytical Hierarchy Process methods (AHP) has been judged the offsite treatment location and incineration to be the best site and best alternative treatment technology for processing infectious waste respectively [17]. Nevertheless Chen et al. 2013 reported, by the end of 2012, in different cities of China 272 modern, high-standard, centralized medical waste disposal facilities installed. Among those facilities about 50% are non-incineration treatment facilities, including the technologies of high temperature steam, chemical disinfection and microwave. In that study Chen et al. concluded each of the non-incineration technologies has its advantages and disadvantages, and none of those technology is not the best alone, due to the complexity of medical waste treatment and disposal [26].

## Conclusion

Practical condition assessment indicated that most of on-site medical waste treating systems had operation and maintenance problems. In additions, other problems like difficulties in preparing spare parts, insufficient financial rescores, could be considered. Analytical Hierarchy Process (AHP) technique demonstrated that off-site treatment of medical waste was in much higher priority than the on-site one. Therefore, based on the result of AHP technique, the current problems and unsuccessful experience with on-site treating facilities, off-site central treatment can be considered as an alternative. It was predicted that the advantages of off-site central systems would be more than their disadvantages. The benefits such as the following ones can be also expected: practicability of accepting and treating medical waste of minor medical producers in the city; feasibility of receiving and treating medical waste of the surrounding cities and villages; more cost-effectiveness for larger units by reducing capital, maintenance and operating costs; more economical preparation of spare capacity, easy performance of future expansion and modification; feasibility of employing private sector capacity in installing and operation of medical waste testing facilities; ensuring more efficient operation of central off-site facilities in comparison with several plants (hospitals' on-site systems) in which skilled workers may not be readily available. Nevertheless in the planning of off-site central treatment facilities, disadvantages such as risks of waste consignment to public health and the environment or illegal recycling should be considered and minimized by strict monitoring and regulation.

An amendment could be made in Iran’s current hazardous waste regulations to have infectious-hazardous waste sent to the central off-site for treatment. To begin with, this plan should be tested and official approval should be received; then, a central off-site can be put into practice as a pilot. Next, if it was practically successful, it could be expanded to other provinces and cities. The investment for off-site facilities can be carried out by privet sector or government. Each producer of medical waste should pay their own waste collection, treatment and disposal costs monthly. Meanwhile, the Health Ministry and the Environmental Protection Agency should strictly supervise medical waste collection, treating, and disposal.

## Competing interests

The authors declare that they have no competing interests.

## Authors’ contributions

The overall implementations of this study were the results of efforts by corresponding author. All authors have made contribution into the review and finalization of this manuscript. All authors read and approved the final manuscript.
